# Supplementary material for: GLP-1R associates with VAPB and SPHKAP at ERMCSs to regulate β-cell mitochondrial remodelling and function
Source: Nat Commun. 2025 Dec 10;16:11010. doi: 10.1038/s41467-025-66115-x (PMC12696101; doi:10.1038/s41467-025-66115-x)
Supplement: Supplementary file 1 — Supplementary Information [file 41467_2025_66115_MOESM1_ESM.pdf]

Supplementary Information

Supplementary Figures

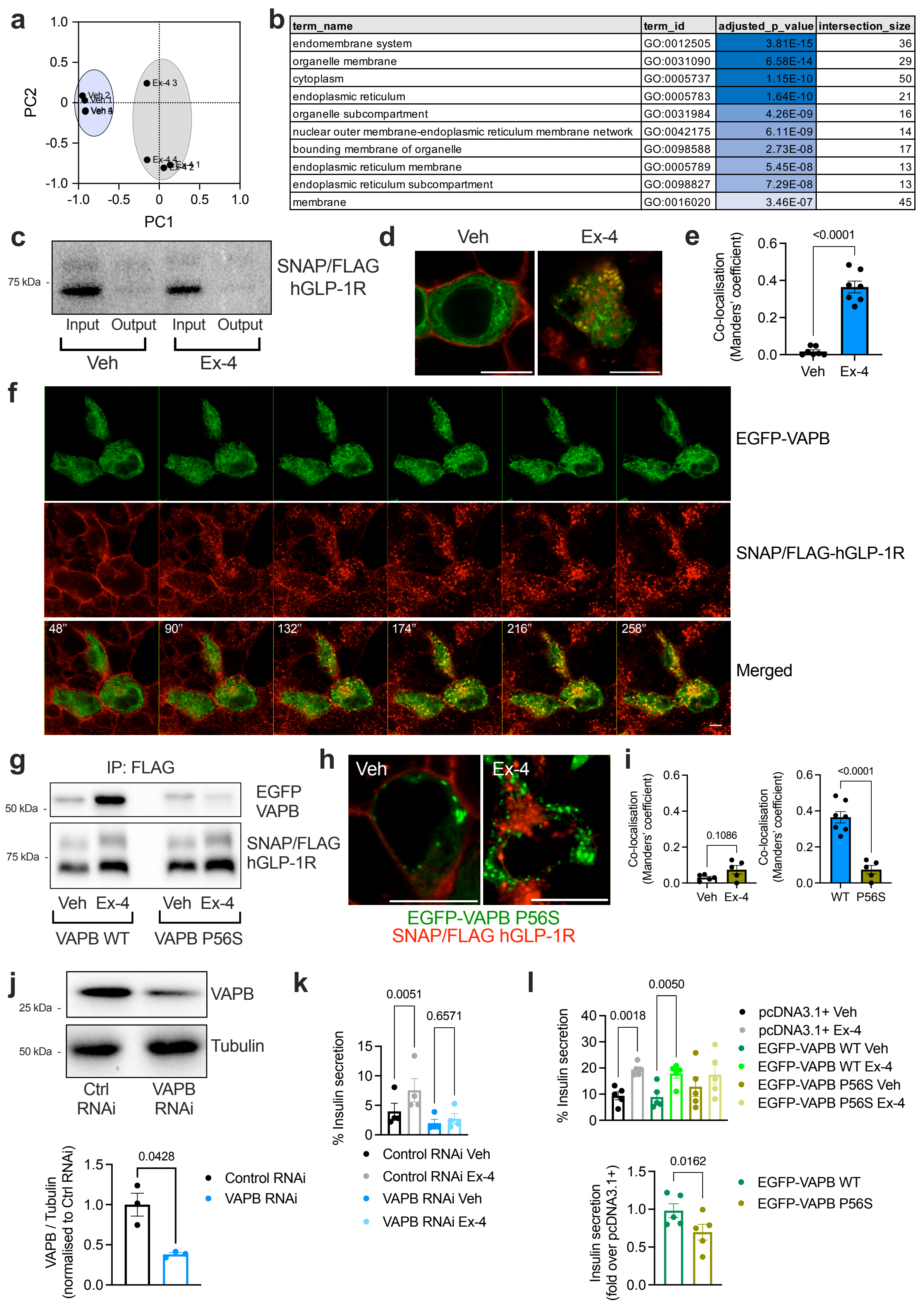

Supplementary Fig. 1. Human GLP-1R β-cell interactome and validation of VAPB-hGLP-1R interaction – Extra data. a, Principal component analysis (PCA) of *n*=4 biological repeats from interactome data from Fig. 1a,b. **b**, Pathway enrichment analysis using gProfiler; results show 10 most significant gene ontology terms represented in the interactome data from Fig.1a,b. **c**, SNAP/FLAG-hGLP-1R levels in input *versus* output supernatants from co-IP experiments from Fig. 1e. **d**, Confocal microscopy analysis of SNAP/FLAG-hGLP-1R (red) co-localisation with EGFP-VAPB (green) in vehicle (Veh, left) *versus* exendin-4 (Ex-4, right)-stimulated INS-1 832/3 SNAP/FLAG-hGLP-1R cells; size bars, 10 µ; images representative of *n*=3 biologically independent experiments. **e**, SNAP/FLAG-hGLP-1R - EGFP-VAPB co-localisation (Mander's coefficient) in Veh *versus* Ex-4-stimulated conditions from data in (**d**); *n*=7 cells from 3 independent experiments, p value as indicated by two-tailed unpaired t-test. **f**, Time-series confocal microscopy analysis of SNAP/FLAG-hGLP-1R (red) and EGFP-VAPB (green) localisation in response to exendin-4 (Ex-4) in INS-1 832/3 SNAP/FLAG-GLP-1R cells; time post-Ex-4 addition indicated; size bar, 5 µm; images representative of *n*=3 biologically independent experiments. **g**, EGFP-VAPB WT *versus* P56S mutant co-IP with SNAP/FLAG-hGLP-1R in vehicle (Veh) *versus* Ex-4-stimulated INS-1 832/3 SNAP/FLAG-hGLP-1R cells; *n*=1. **h**, Confocal microscopy analysis of SNAP/FLAG-hGLP-1R (red) co-localisation with EGFP-VAPB WT or P56S (green) in Veh *versus* Ex-4-stimulated INS-1 832/3 SNAP/FLAG-hGLP-1R cells; size bars, 5 µm; images representative of *n*=3 biologically independent experiments. **i**, SNAP/FLAG-hGLP-1R - EGFP-VAPB P56S co-localisation (Mander's coefficient) in Veh *versus* Ex-4-stimulated conditions from data in (**h**) (left), and comparison of SNAP/FLAG-hGLP-1R co-localisation with EGFP-VAPB WT *versus* P56S following Ex-4 stimulation (right); *n*=5-7 cells from 3 independent experiments, p values as indicated by two-tailed unpaired t-test. **j**, Western blot analysis of endogenous VAPB levels in Control *versus* VAPB RNAi-treated INS-1 832/3 cells; representative blots and quantification of VAPB knockdown shown; *n*=3 biologically independent experiments, p value as indicated by two-tailed ratio paired t-test. **k**, Ex-4-induced potentiation of insulin secretion in Control *versus* VAPB RNAi-treated INS-1 832/3 cells; *n*=4 biologically independent experiments, p values as indicated by one-way ANOVA with Šidák post-hoc test. **l**, Ex-4-induced insulin secretion in pcDNA3.1+, EGFP-VAPB WT and P56S-transfected INS-1 832/3 cells; fold over pcDNA3.1+ also shown, *n*=5 biologically independent experiments; p values as indicated by one-way ANOVA with Šidák post-hoc test, or by two-tailed paired t-test. Data are mean ± SEM.

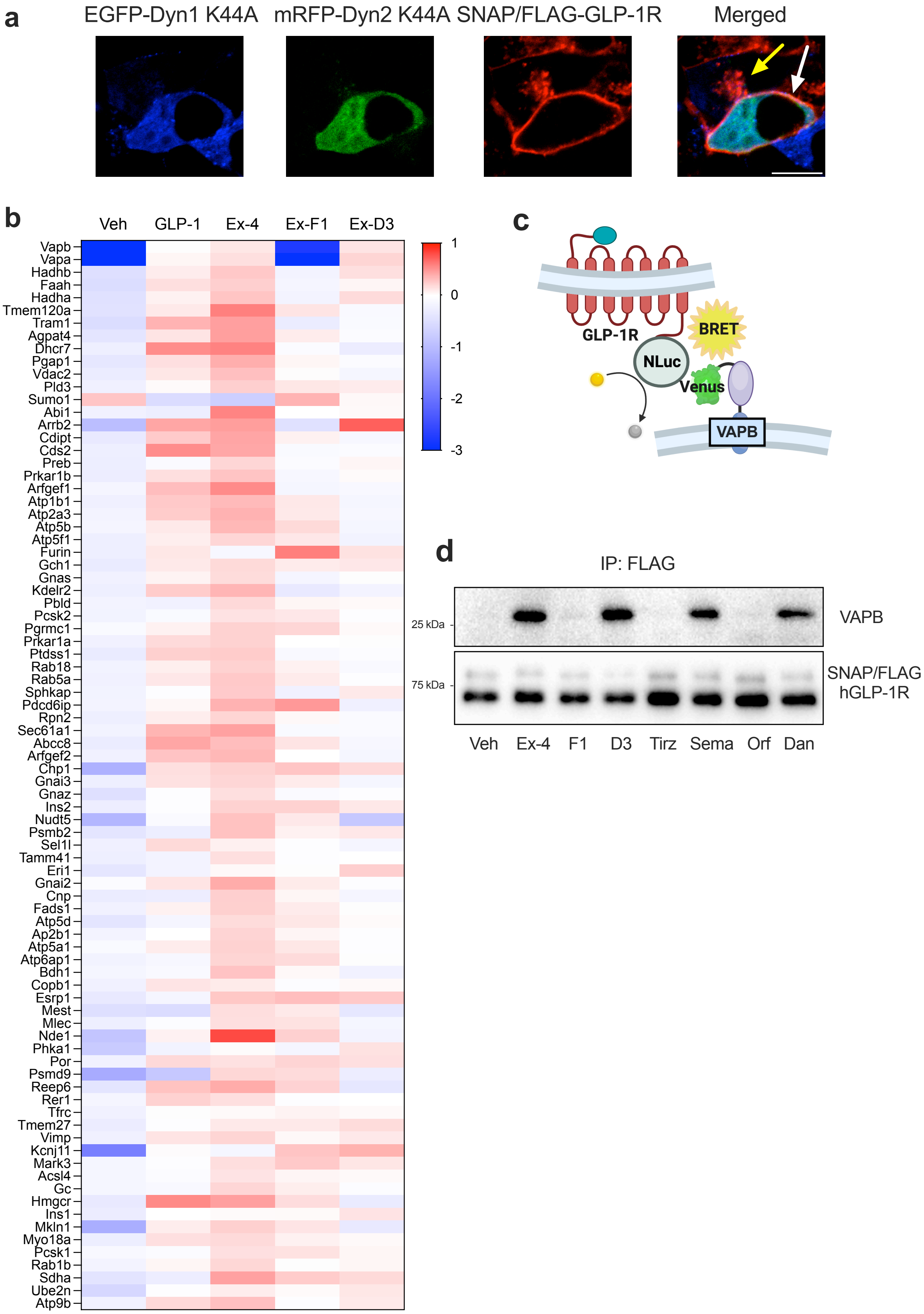
a
EGFP-Dyn1 K44A
mRFP-Dyn2 K44A
SNAP/FLAG-GLP-1R
Merged
b
Veh
GLP-1
Ex-4
Ex-F1
Ex-D3
Vapb
Vapa
Hadhb
Faah
Hadha
Tmem120a
Tram1
Agpat4
Dhcr7
Pgap1
Vdac2
Pld3
Sumo1
Abi1
Arrb2
Cdipt
Cds2
Preb
Prkar1b
Arfgef1
Atp1b1
Atp2a3
Atp5b
Atp5f1
Furin
Gch1
Gnas
Kdelr2
Pbld
Pcsk2
Pgrmc1
Prkar1a
Ptdss1
Rab18
Rab5a
Sphkap
Pdcd6ip
Rpn2
Sec61a1
Abcc8
Arfgef2
Chp1
Gnai3
Gnaz
Ins2
Nudt5
Psmb2
Sel1l
Tamm41
Eri1
Gnai2
Cnp
Fads1
Atp5d
Ap2b1
Atp5a1
Atp6ap1
Bdh1
Copb1
Esrp1
Mest
Mlec
Nde1
Phka1
Por
Psmd9
Reep6
Rer1
Tfrc
Tmem27
Vimp
Kcnj11
Mark3
Acsl4
Gc
Hmgcr
Ins1
Mkln1
Myo18a
Pcsk1
Rab1b
Sdha
Ube2n
Atp9b
1
0
-1
-2
-3
c
GLP-1R
BRET
NLuc
Venus
VAPB
d
IP: FLAG
25 kDa
VAPB
75 kDa
SNAP/FLAG
hGLP-1R
Veh
Ex-4
F1
D3
Tirz
Sema
Orf
Dan

Supplementary Fig. 2. Internalisation-dependent hGLP-1R-VAPB binding – Extra data. **a**, Confocal microscopy analysis of SNAP/FLAG-hGLP-1R localisation in exendin-4 (Ex-4)-stimulated INS-1 832/3 SNAP/FLAG-hGLP-1R cells co-expressing dominant negative dynamin (Dyn)1/2 K44A; white arrow indicates plasma membrane SNAP/FLAG-hGLP-1R localisation with Dyn1/2 K44A co-expression; yellow arrow indicates endosomal SNAP/FLAG-hGLP-1R localisation in a non-Dyn1/2 K44A-expressing cell; size bar, 5 µm; images representative of *n*=3 biologically independent experiments. **b**, Heatmap of GLP-1R interactor enrichment analysis from anti-FLAG co-immunoprecipitates of INS-1 832/3 SNAP/FLAG-hGLP-1R cells stimulated for 5 minutes with the indicated agonists; blue, decreased; red, increased enrichment; LC-MS/MS data analysed in LFQ-Analyst and normalised to hGLP-1R levels for each experimental sample; colour scale centred around overall median value; *n*=4 biologically independent experiments for exendin-4 (Ex-4), exendin-F1 (Ex-F1) and exendin-D3 (Ex-D3) and *n*=3 biologically independent experiments for GLP-1. **c**, Schematic of the hGLP-1R-Nanoluciferase (NLuc) – Venus-VAPB interaction NanoBRET assay. Created in BioRender. Tomas, A. (2025) https://BioRender.com/3u1mqeg. **d**, VAPB co-IP with SNAP/FLAG-hGLP-1R in INS-1 832/3 SNAP/FLAG-hGLP-1R cells stimulated for 5 minutes with: vehicle (Veh), Ex-4, exendin-F1 (Ex-F1), exendin-D3 (Ex-D3), tirzepatide (Tirz), semaglutide (Sema) all used at 100 nM; orforglipron (Orf) and danuglipron (Dan) used at 5 µM; blots representative of *n*=3 biologically independent experiments.

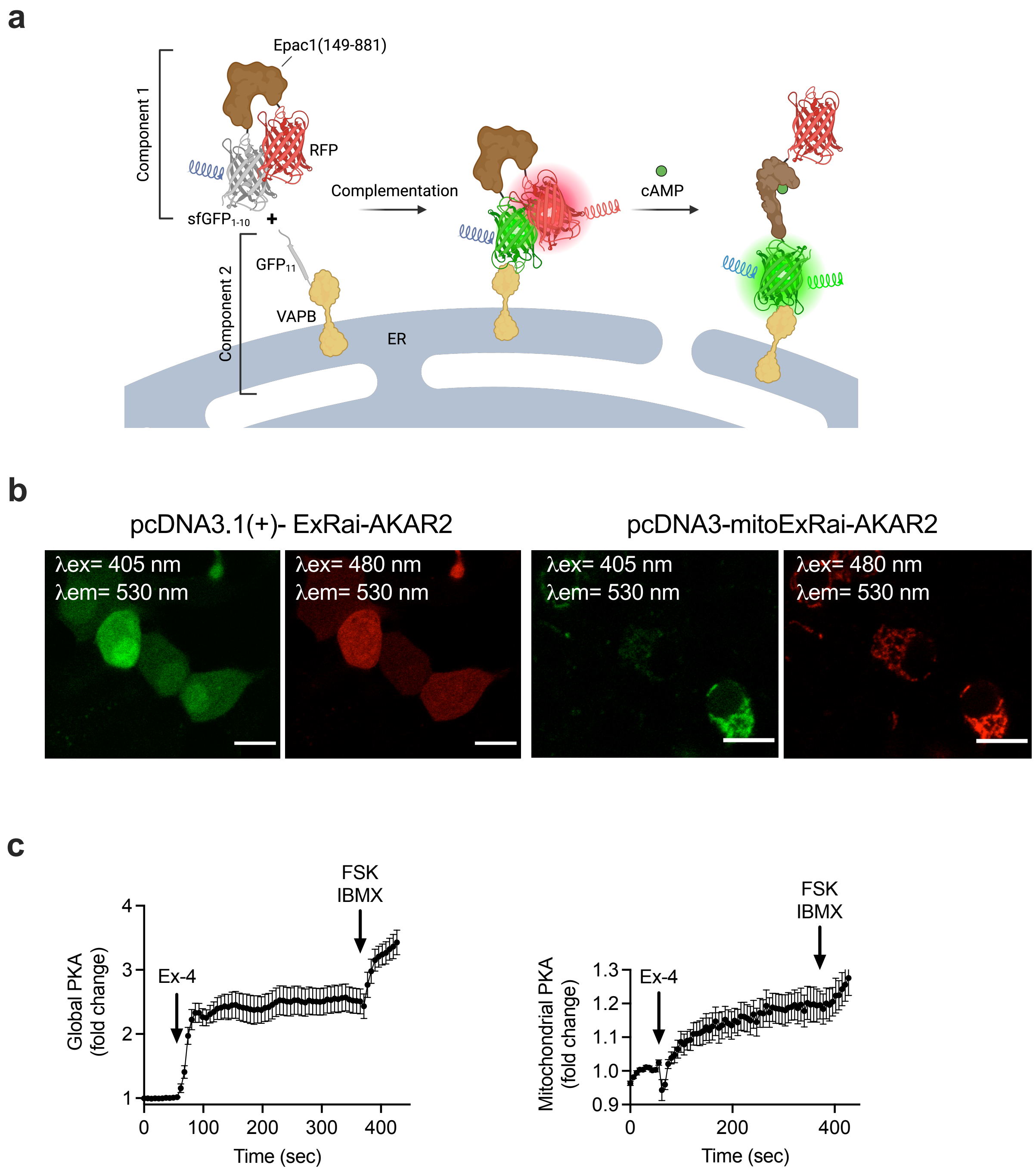

Supplementary Fig. 3. VAPB-localised GLP-1R signalling – Extra data. a, Schematic of the FluoSTEP method used to quantify GLP-1R-induced VAPB-localised cAMP generation; Epac1 (149-881), exchange protein activated by cAMP, amino acids 149-881; RFP, red fluorescent protein; sfGFP$_{1-10}$, superfolder green fluorescent protein helices 1-10; GFP$_{11}$, 11th helix of sfGFP; VAPB, vesicle-associated membrane protein-associated protein B. Created in BioRender. Tomas, A. (2025) https://BioRender.com/4a6wd09. **b**, Localisation of the excitation-ratiometric global PKA biosensor pcDNA3.1(+)-ExRai-AKAR2 and mitochondrial PKA biosensor pcDNA3-mitoExRai-AKAR2 in INS-1 832/3 cells; size bars,10 µm; *n*=1. **c**, Ex-4-stimulated global and mitochondrial PKA responses in INS-1 832/3 cells, measured with the biosensors from (**b**); Forskolin (FSK) + IBMX added to record maximal responses; *n*=10 cells from 1 biological repeat. Data are mean ± SEM.

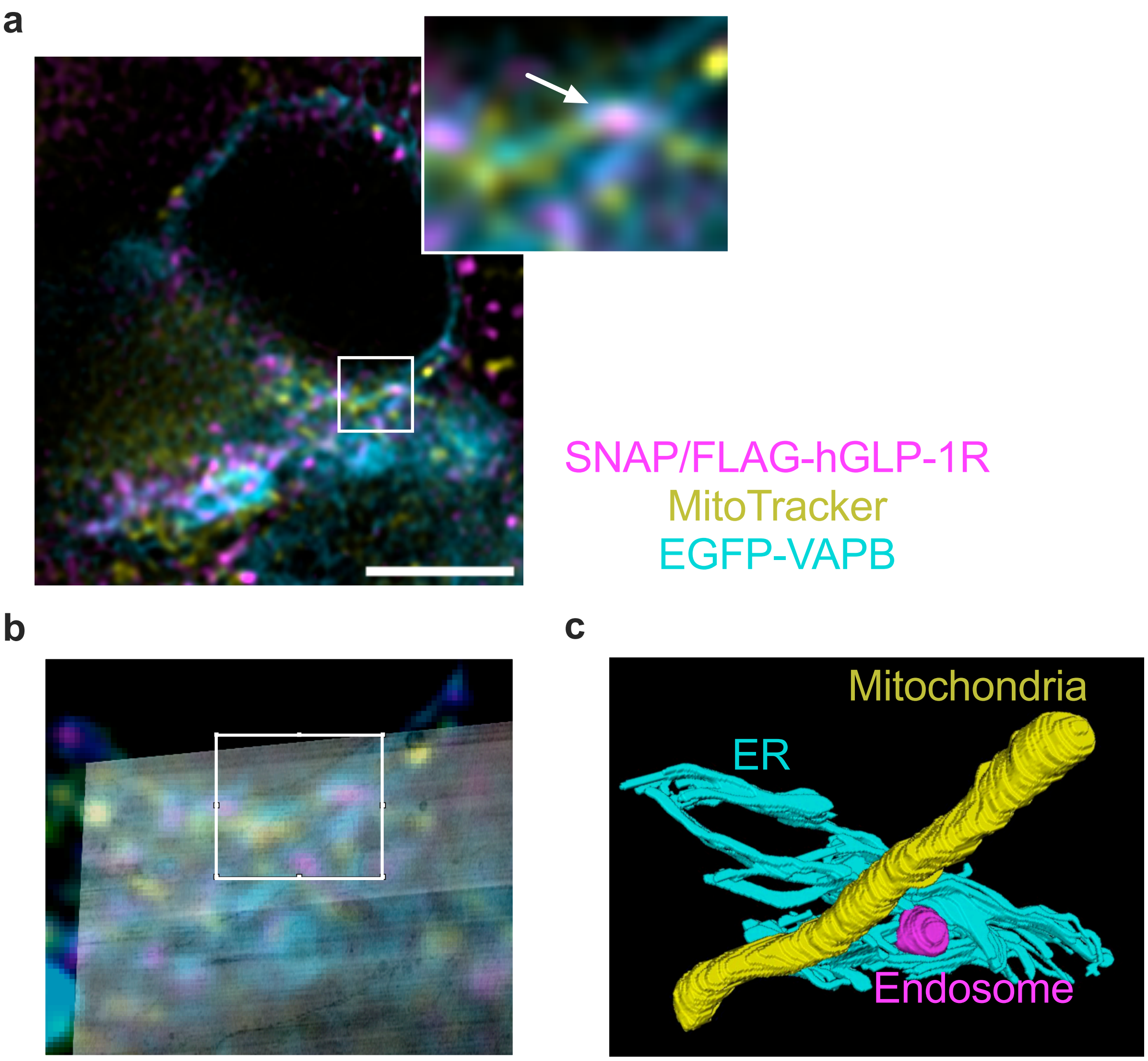

Supplementary Fig. 4. Human GLP-1R localisation to ERMCSs – Extra data. a, Single cryo-confocal slice from an exendin-4 (Ex-4)-stimulated INS-1 832/3 SNAP/FLAG-hGLP-1R cell, with signals for EGFP-VAPB (cyan), SNAP/FLAG-hGLP-1R (magenta), and mitochondria (yellow); inset, magnified area; arrow points to SNAP/FLAG-hGLP-1R-positive endosome used for cryo-CLEM analysis; size bar, 5 µm. **b**, Overlay of a cryo-confocal slice aligned with the corresponding single slice of a resliced 3D cryo-FIB-SEM volume. **c**, 3D rendering of the segmentation of the cryo-FIB-SEM volume data from (**b**), with ER (cyan), mitochondria (yellow) and SNAP/FLAG-hGLP-1R-positive endosome (magenta) shown. Created in Icy.

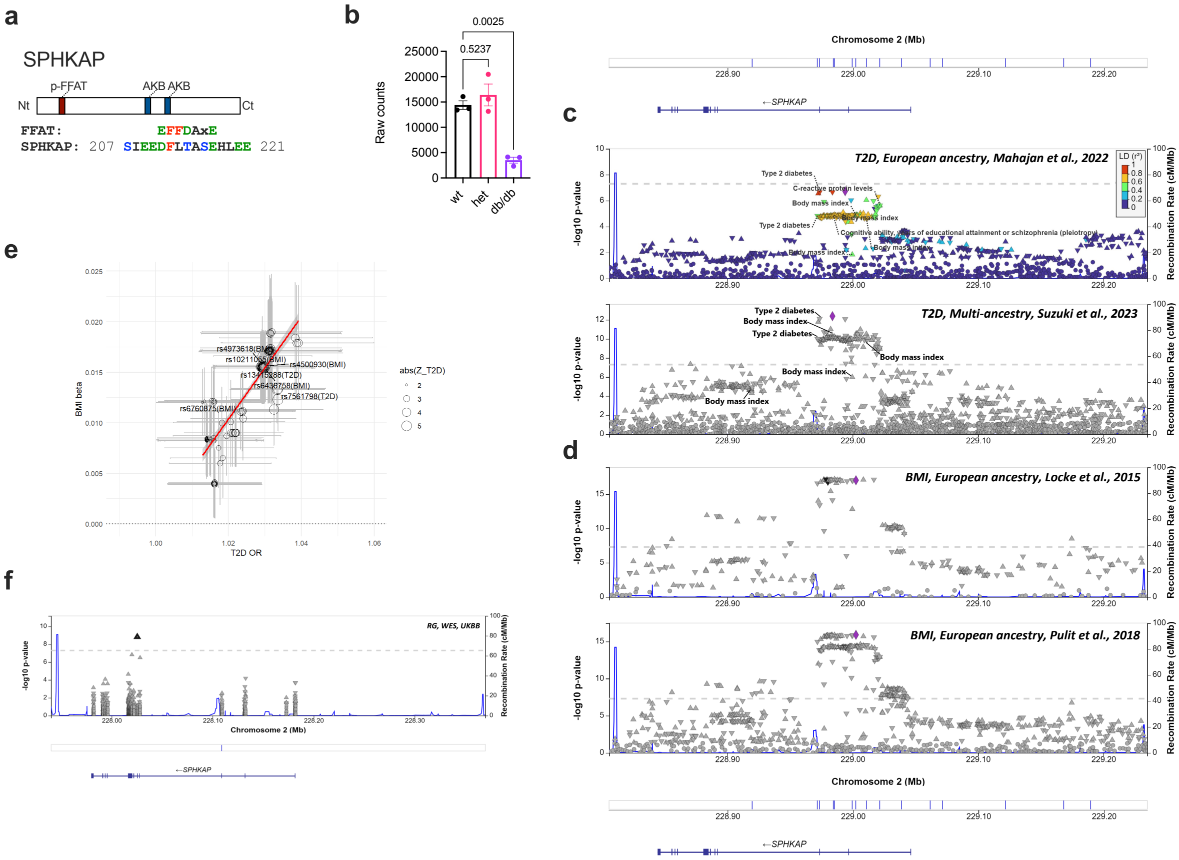

Supplementary Fig. 5. Gene expression data and genetic association of *SPHKAP* variants with T2D and BMI. a, Schematic of the SPHKAP protein primary structure. AKB, A-kinase binding domain; pFFAT, phospho-FFAT motif. Alignment of SPHKAP FFAT motif to the canonical FFAT sequence depicted; alcoholic residues are highlighted in blue, acidic residues in green, and aromatic residues in red; all other residues are in black. **b**, Analysis of *SPHKAP* mRNA levels from primary islets isolated from WT *versus* heterozygous and homozygous *db*/*db* mice; data obtained from RNA-Seq dataset GSE107489[1]; *n*=3 biologically independent experiments, p values as indicated by one-way ANOVA with Dunnett post-hoc test. Data are mean ± SEM. **c**, *SPHKAP* gene region variant associations from T2D European ancestry[2] and multi-ancestry[3] GWAS; y-axes represent significance (-$\log_{10}$ p-value) of phenotype–variant associations and x-axes genomic location of the variants (NCBI human genome build 37). Secondary y-axes represent recombination fraction from 1000 Genomes Project and horizontal dashed lines highlight association threshold for significance ($p=5\times10^{-8}$). Lead variant with the highest significance is shown as a purple diamond and correlation (LD, r^2) of other SNPs in the lead variant region represented with colours as per box legend. Variants previously reported for associations with phenotypes in GWAS catalog (www.ebi.gwas.org) are indicated with respective phenotypes listed. **d**, As for (**c**) for BMI GWAS meta-analyses[4, 5]. **e**, Comparison of the effects of *SPHKAP* gene region variants showing at least nominal association (p<0.05) with both T2D risk and high adiposity (BMI) from GWAS meta-analyses from (**c**,**d**). X-axis [odds ratio (OR) with 95% confidence interval] corresponds to associations with T2D, while y-axis (β with standard error) corresponds to associations with BMI. Variant points are scaled in size to their significance with T2D (z-score from association test). Seven *SPHKAP* single nucleotide polymorphisms (SNPs), 2 for T2D and 5 for high BMI, identified from GWAS Catalog (www.ebi.gwas.org) as being in high linkage disequilibrium (LD) with each other, are indicated. The fitted red regression line indicates Pearson correlation between BMI and T2D-associated signals at the *SPHKAP* locus. **f**, *SPHKAP* coding variant effects on random glucose (RG) from UK Biobank (UKBB) whole-exome sequencing (WES) data for ~450,000 individuals; y-axis represents significance (-$\log_{10}$ p-value) of variant association and

x-axis represents the genomic location of variants (NCBI human genome build 38). Secondary y-axis represents recombination fraction from 1000 Genomes Project and horizontal dashed line highlights the association threshold for significance ($p=5\times10^{-8}$). RG levels in mmol/L are natural logarithm transformed and adjusted for time since last meal (t) as $t+t^2+t^3$, age, sex, and six principal components (derived from UKBB GWAS data).

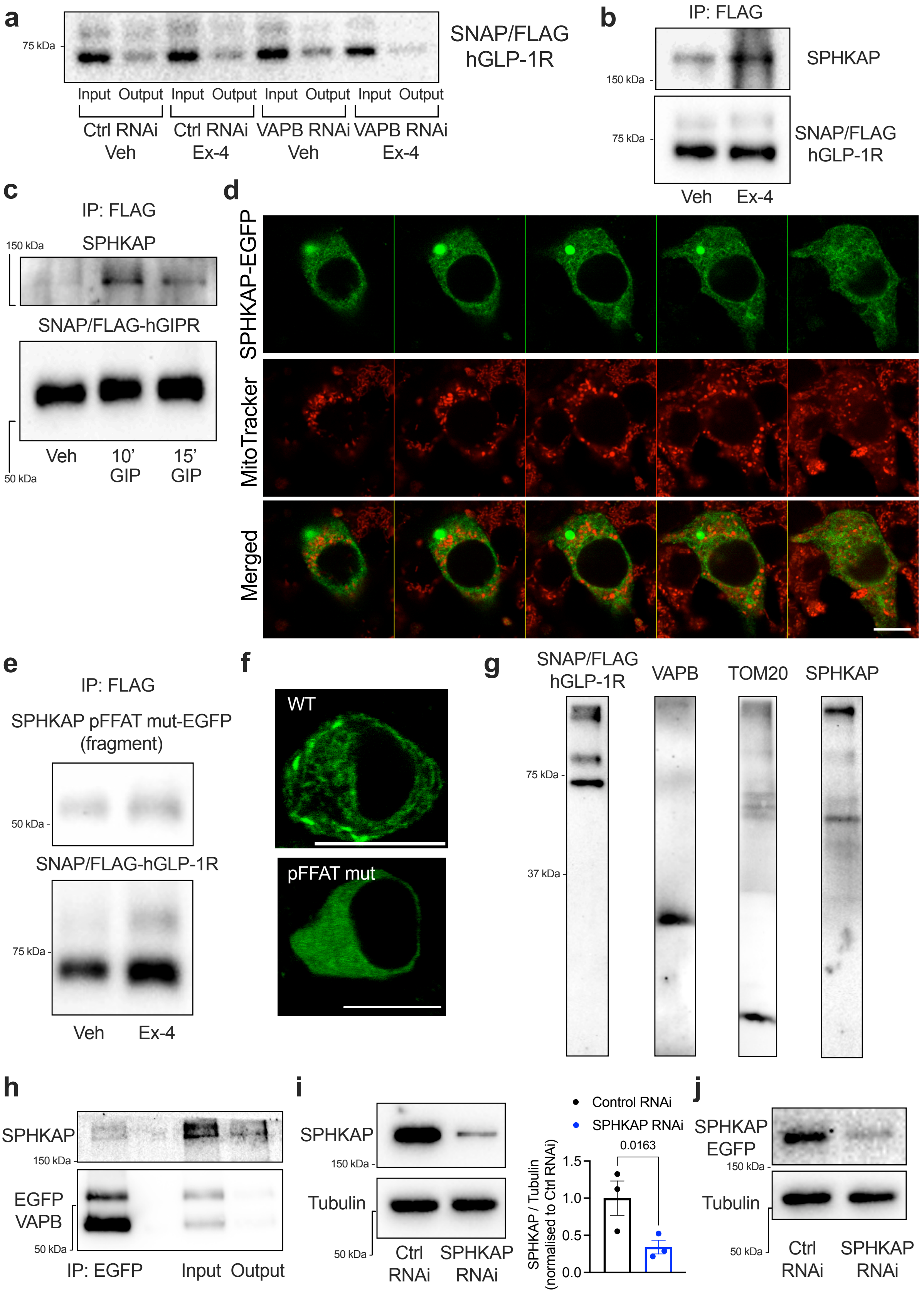

Supplementary Fig. 6. Human GLP-1R–SPHKAP association via shared VAPB binding – Extra data. a, SNAP/FLAG-hGLP-1R levels in input *versus* output supernatants from co-IP experiments from Fig. 4a,b. **b**, Endogenous SPHKAP co-IP with SNAP/FLAG-hGLP-1R in

vehicle (Veh) *versus* exendin-4 (Ex-4)-stimulated INS-1 832/3 SNAP/FLAG-hGLP-1R cells; blots representative of *n*=2 biologically independent experiments. **c**, Endogenous SPHKAP co-IP with SNAP/FLAG-hGIPR in Veh *versus* GIP-stimulated INS-1 832/3 SNAP/FLAG-hGIPR cells; *n*=1. **d**, Z-stack series of confocal microscopy images of SPHKAP-EGFP and mitochondria (imaged with MitoTracker Red) in INS-1 832/3 cells; size bar, 10 µm; images representative of *n*=3 biologically independent experiments. **e**, SPHKAP-EGFP pFFAT motif mutant (DFLTESE) co-IP with SNAP/FLAG-hGLP-1R in Veh *versus* Ex-4-stimulated INS-1 832/3 SNAP/FLAG-hGLP-1R cells; *n*=1; GFP-positive fragment detected due to intrinsic instability of co-IPed full-length fusion protein. **f**, Confocal microscopy analysis of SPHKAP-EGFP WT *versus* pFFAT motif mutant in INS-1 832/3 cells; size bars, 5 µm; images representative of *n*=3 biologically independent experiments. **g**, Western blot analysis of SNAP/FLAG-hGLP-1R, VAPB, and SPHKAP in Tom20-positive MAM fraction purified from Ex-4-stimulated INS-1 832/3 SNAP/FLAG-hGLP-1R cells; *n*=1. **h**, Endogenous SPHKAP - EGFP-VAPB co-IP in INS-1 832/3 cells, including input and output fractions; *n*=1. **i**, Western blot analysis of endogenous SPHKAP levels in Control *versus* SPHKAP RNAi-treated INS-1 832/3 cells. Quantification of SPHKAP knockdown also shown; *n*=3 biologically independent experiments, p value as indicated by two-tailed ratio paired t-test. Data are mean ± SEM. **j**, Western blot analysis of SPHKAP-EGFP levels in Control *versus* SPHKAP RNAi-treated INS-1 832/3 cells; *n*=1.

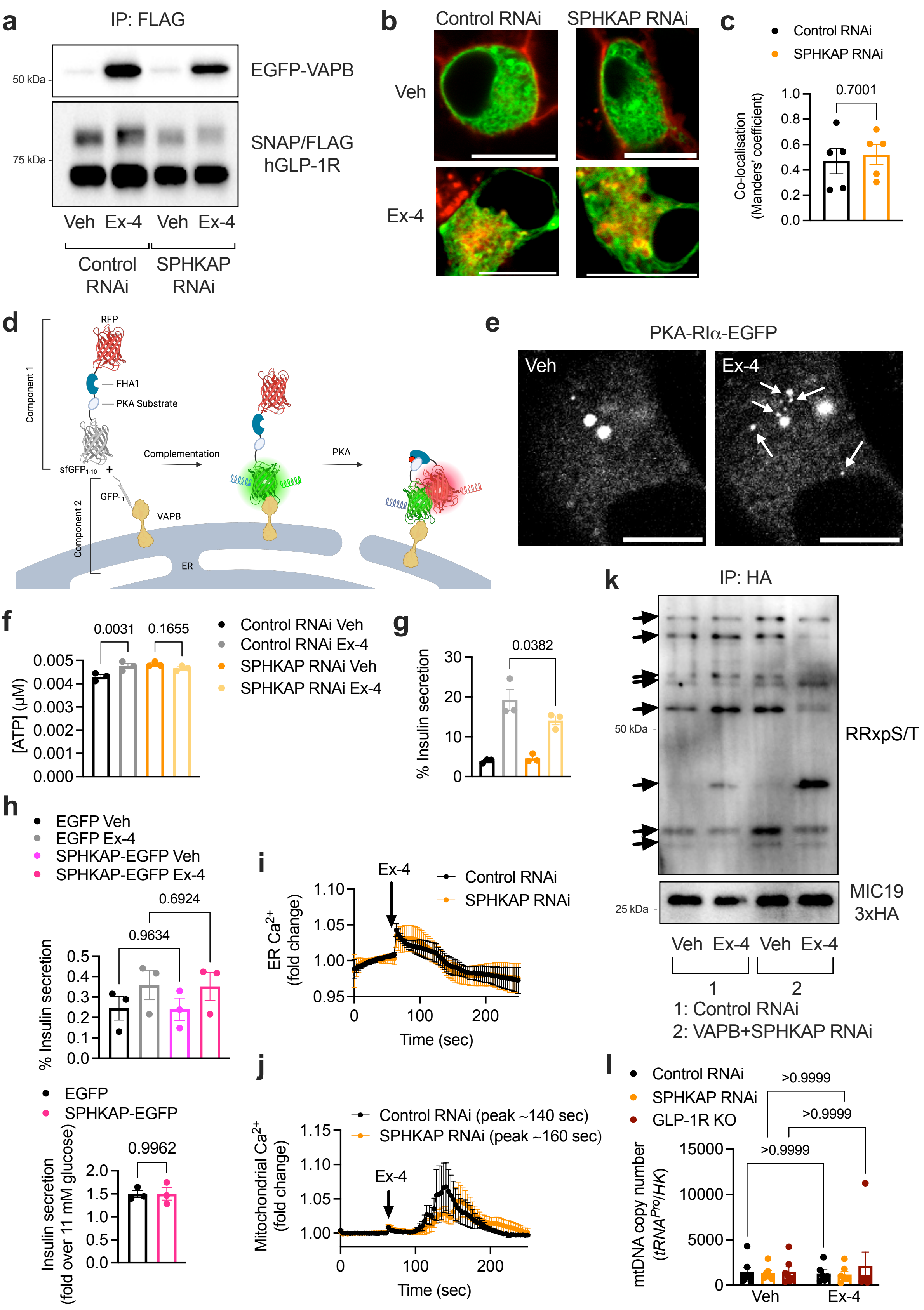

Supplementary Fig. 7. Regulation of GLP-1R function by SPHKAP – Extra data. a, EGFP-VAPB co-IP with SNAP/FLAG-hGLP-1R in Control *versus* SPHKAP RNAi-treated INS-1 832/3 SNAP/FLAG-hGLP-1R cells under vehicle (Veh) *versus* exendin-4 (Ex-4)-stimulated

conditions; *n*=1. **b**, Confocal microscopy analysis of EGFP-VAPB co-localisation with SNAP/FLAG-hGLP-1R in Veh *versus* Ex-4-stimulated conditions in Control *versus* SPHKAP RNAi-treated INS-1 832/3 SNAP/FLAG-hGLP-1R cells; size bars, 10 μm; images representative of *n*=3 biologically independent experiments. **c**, SNAP/FLAG-hGLP-1R - EGFP-VAPB co-localisation (Mander's coefficient) in Ex-4-stimulated Control *versus* SPHKAP RNAi-treated INS-1 832/3 SNAP/FLAG-hGLP-1R cells from data in (**b**); *n*=5 cells from 3 independent experiments, p values as indicated by two-tailed unpaired t-test. **d**, Schematic of the FluoSTEP method used to quantify GLP-1R-induced VAPB-localised PKA activity; FHA1, forkhead-associated 1 phospho-peptide binding protein; PKA, protein kinase A; RFP, red fluorescent protein; $sfGFP_{1-10}$, superfolder green fluorescent protein helices 1-10; GFP_{11}, 11th helix of sfGFP; VAPB, vesicle-associated membrane protein-associated protein B. Created in BioRender. Zhu, M. (2025) https://BioRender.com/csuvuf4. **e**, Confocal microscopy analysis of PKA-RIα-EGFP localisation in Veh *versus* Ex-4-stimulated INS-1 832/3 cells, size bars, 10 μm; arrows indicate newly formed PKA-RIα-EGFP puncta; images representative of *n*=3 biologically independent experiments. **f**, Ex-4-induced changes in ATP level in Control *versus* SPHKAP RNAi-treated INS-1 832/3 cells; *n*=3 biologically independent experiments, p values as indicated by one-way ANOVA with Šidák post-hoc test. **g**, Ex-4-induced potentiation of insulin secretion in Control *versus* SPHKAP RNAi-treated INS-1 832/3 cells; *n*=3 biologically independent experiments, p value as indicated by one-way ANOVA with Šidák post-hoc test. **h**, Ex-4-induced potentiation of insulin secretion in INS-1 832/3 cells transfected with EGFP *versus* SPHKAP-EGFP; *n*=3 biologically independent experiments; p values as indicated by one-way ANOVA with Šidák post-hoc test; fold over 11 mM glucose also shown; p value as indicated by two-tailed paired t-test. **i**, Ex-4 induced changes in ER Ca^{2+}, measured with pCAG G-CEPIA1er, in Control *versus* SPHKAP RNAi-treated INS-1 832/3 cells; *n*=4 biologically independent experiments. **j**, As in (**i**) for mitochondrial Ca^{2+}, measured with pCAG mito-RCaMP1h; peak response times indicated per condition; *n*=4 biologically independent experiments. **k**, PKA-dependent phosphorylation of MICOS complex components isolated by MIC19-3xHA co-IP in Control *versus* VAPB+SPHKAP RNAi-treated INS-1 832/3 cells under Veh *versus* Ex-4-stimulated conditions, determined by Western blotting with an anti-RRx-pS/T antibody; arrows indicate proteins showing changes in PKA-dependent phosphorylation; *n*=1. **l**, Mitochondrial DNA (mtDNA) copy number, measured as a ratio of mitochondrial $tRNA^{Pro}$ over hexokinase (*HK*) in Veh *versus* Ex-4-stimulated Control and SPHKAP RNAi-treated INS-1 832/3 cells, as well as in INS-1 832/3 GLP-1R KO cells; *n*=7 biologically independent experiments; p values as indicated by two-way ANOVA with Tukey post-hoc test. Data are mean ± SEM.

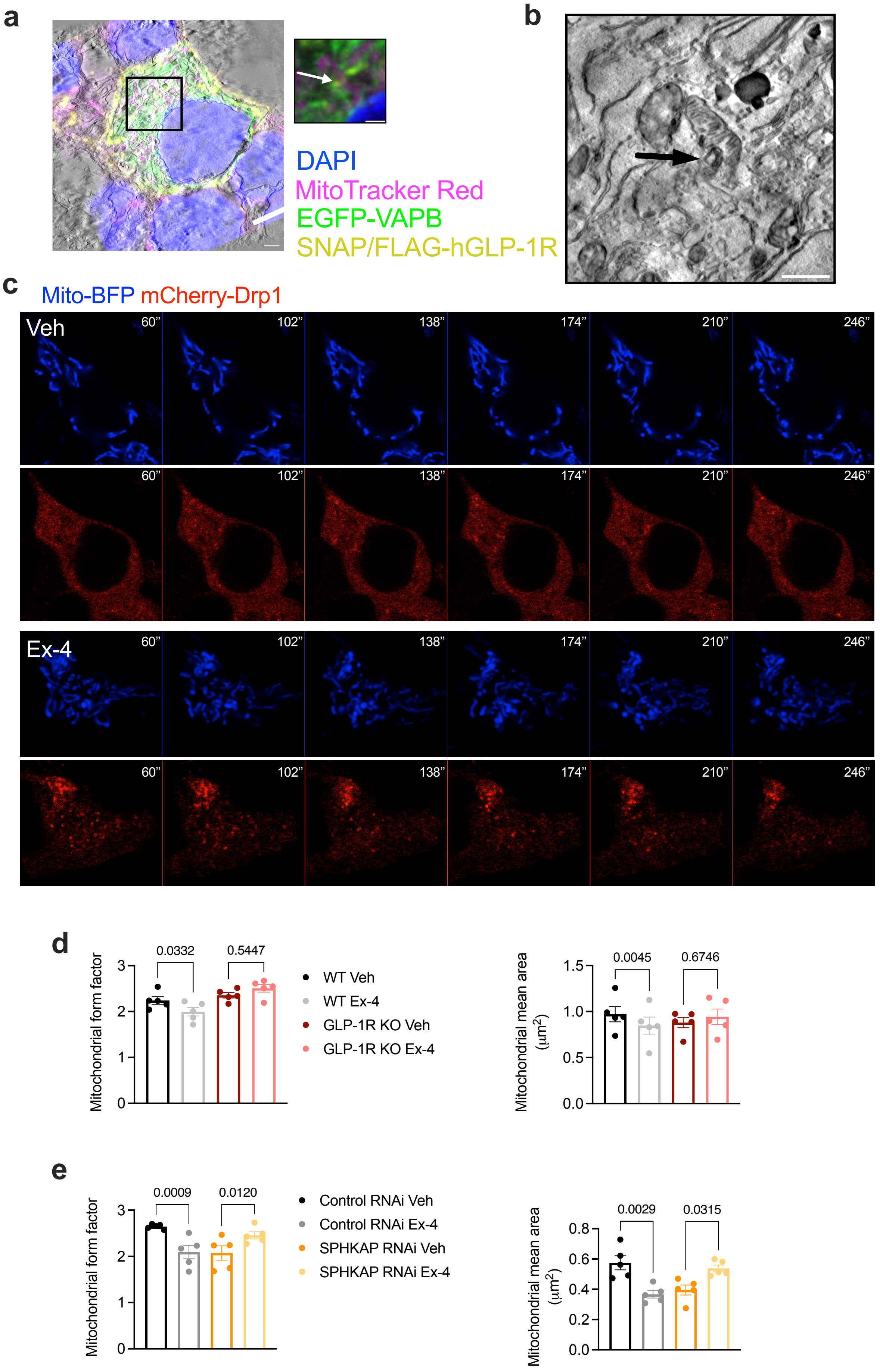
a
DAPI
MitoTracker Red
EGFP-VAPB
SNAP/FLAG-hGLP-1R
b
c
Mito-BFP mCherry-Drp1
Veh
60"
102"
138"
174"
210"
246"
Ex-4
d
Mitochondrial form factor
0.0332
0.5447
WT Veh
WT Ex-4
GLP-1R KO Veh
GLP-1R KO Ex-4
Mitochondrial mean area (μm²)
0.0045
0.6746
e
0.0009
0.0120
Control RNAi Veh
Control RNAi Ex-4
SPHKAP RNAi Veh
SPHKAP RNAi Ex-4
0.0029
0.0315

Supplementary Fig. 8. GLP-1R and SPHKAP-dependent mitochondrial remodelling – Extra data. a, Overlay of a single confocal slice, with signals for VAPB (green), GLP-1R (yellow), mitochondria (magenta), and DAPI (blue) shown, and corresponding single slice of an EM tomogram following CLEM analysis of exendin-4 (Ex-4)-stimulated INS-1 832/3 SNAP/FLAG-hGLP-1R cells; size bar, 1 μm; magnified confocal slice region with arrow pointing to SNAP/FLAG-hGLP-1R positive signal also shown; size bar, 1 μm. **b**, Magnification of tomogram slices corresponding to magnified confocal slice from (**a**); arrow points to the same SNAP/FLAG-hGLP-1R-positive lysosome as in (**a**) embedded in a mitochondrion, indicating mitophagy; size bar, 1 μm. **c**, Single channel images corresponding to mito-BFP (blue) and mCherry-Drp1 (red) signals from Fig. 5g. **d**, Quantification of mitochondrial mean form factor and mean area in Veh *versus* Ex-4-stimulated WT and GLP-1R KO INS-1 832/3 cells; *n*=5 biologically independent experiments, p values as indicated by one-way ANOVA with Šidák post-hoc test. **e**, As in (**d**) in Veh *versus* Ex-4-stimulated Control *versus* SPHKAP RNAi-treated INS-1 832/3 cells; *n*=5 biologically independent experiments, p values as indicated by one-way ANOVA with Šidák post-hoc test. Data are mean ± SEM.

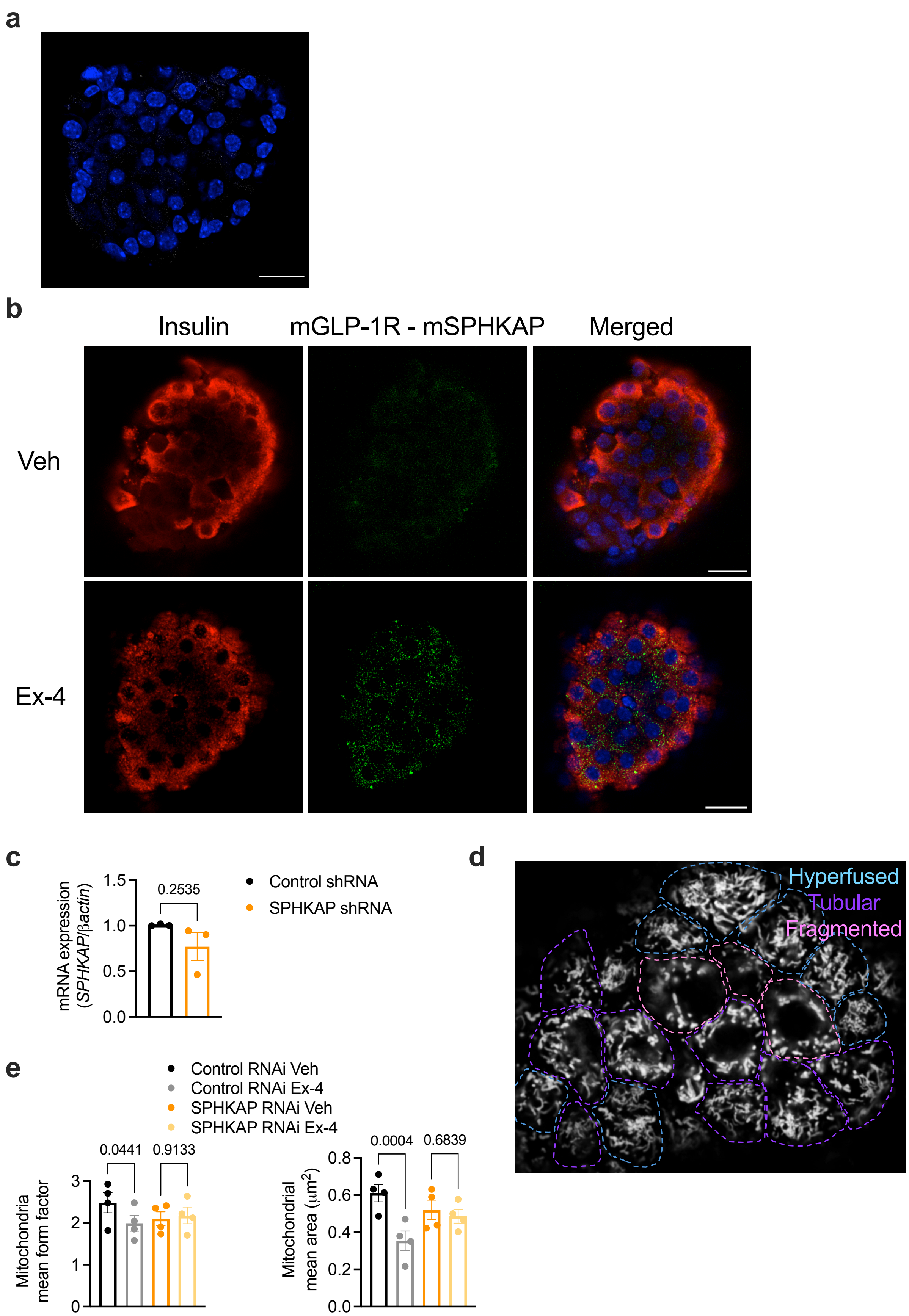

Supplementary Fig. 9. GLP-1R–VAPB/SPHKAP interactions and GLP-1R-dependent mitochondrial remodelling in primary islets – Extra data. a, Negative control (no primary

antibody) for PLA data shown in Fig. 8a; nuclei (DAPI), blue; size bar, 20 µm. **b**, PLA showing interactions between endogenous GLP-1R and SPHKAP (green) in vehicle (Veh) and exendin-4 (Ex-4)-stimulated primary mouse islets with insulin co-staining (red); nuclei (DAPI), blue; size bars, 20 µm; *n*=1. **c**, Quantification of *SPHKAP* mRNA levels by qPCR in Control *versus* SPHKAP shRNA-transduced intact primary mouse islets; *n*=3 biologically independent experiments, p value as indicated by two-tailed paired t-test. **d**, Example of cell segmentation into hyperfused (blue), tubular (purple) or fragmented (pink) mitochondrial phenotypes in a primary mouse islet. **e**, Quantification of mitochondrial mean form factor and mean area in Control *versus* SPHKAP RNAi-treated dispersed mouse islet cells under Veh *versus* Ex-4-stimulated conditions; *n*=4 biologically independent experiments, p values as indicated by one-way ANOVA with Šidák post-hoc test. Data are mean ± SEM.

Supplementary Tables

Name	Origin	Assay
EGFP-VAPB	Gift from Dr Emily Eden, UCL	Co-Immunoprecipitation, confocal microscopy
Venus-VAPB	Generated *in house*	NanoBRET
EGFP-VAPB P56S	Generated *in house*	Co-immunoprecipitation, insulin secretion
GLP-1R-NanoLuc	Generated *in house*	NanoBRET
pcDNA3.1+		Control empty plasmid
Dynamin1 K44A-EGFP	Addgene #34681	Co-Immunoprecipitation, confocal microscopy
Dynamin2 K44A-mRFP	Addgene #128153	Co-Immunoprecipitation, confocal microscopy
SPHKAP-EGFP	Gift from Prof John D. Scott, Howard Hughes Medical Institute[6]	Co-Immunoprecipitation, confocal microscopy
SPHKAP pFFAT mut (DFLTESE)-EGFP	Generated *in house*	Co-Immunoprecipitation, confocal microscopy
pcDNA3-mitoExRai-AKAR2	Addgene #161757	Mitochondrial PKA
pcDNA3.1(+)-ExRai-AKAR2	Addgene #161753	Global PKA
cyto-Ruby3-iATPSnFR1.0	Addgene #102551	ATP
MIC19-3xHA	Gift from Prof Toshihiko Oka, Rikkyo University[7]	Immunoprecipitation, PKA-dependent phosphorylation
SPLICS Mt-ER Long P2A	Addgene #164107	Confocal microscopy of ERMCSs
Proinsulin-NLuc	Addgene #62057	Insulin secretion
mKeima-Red-Mito-7	Addgene #56018	Mitophagy
pCAG G-CEPIA1er	Addgene #105012	ER calcium
pCAG mito-RCaMP1h	Addgene #105013	Mitochondrial calcium
pEGFPC1-hVAP-B	Addgene #104448	Cloning of pcDNA3-GFP11(x7)-VAPB

pcDNA3-GFP11(x7)-Actin	Addgene #181967	Cloning of pcDNA3-GFP11(x7)-VAPB
pcDNA3-GFP11(x7)-VAPB	Generated *in house*	FluoSTEPs assays
mito-BFP	Addgene #49151	Mitochondrial fission
mCh-Drp1	Addgene #49152	Mitochondrial fission
pcDNA3.1(+)-FluoSTEP-ICUE	Addgene #181845	FluoSTEP cAMP
pcDNA3.1(+)-FluoSTEP-AKAR	Addgene #181844	FluoSTEP PKA
4xMTS-Halo	Gift from the Avezov group, UK DRI	Mitochondrial imaging for splitFAST data
ER-RspA-NFAST and OMM-short-RspA-CFAST	Gifts from the Filadi lab, CNR Institute of Neuroscience[8]	ERMCS imaging for splitFAST data
pcDNA3.1(+)-RIα-EGFP	Addgene #181840	Visualisation of PKA-RIα-EGFP biomolecular condensates

Supplementary Table 1. Plasmid cDNA origin and use.

Name	Origin
Control siRNA	Ambion™ Silencer™ Select Negative Control #1 siRNA Cat#:4390843
VAPB siRNA	Ambion™ Silencer™ Select Pre-Designed siRNA Cat#:4390771, s133396
SPHKAP siRNA	Ambion™ Silencer™ Select Pre-Designed siRNA Cat#:4390771, s164929

Supplementary Table 2. siRNA origin.

DATE OF ARRIVAL	BMI	SOURCE
**14.12.23**	24.4	CEED
**29.01.24**	29.75	CEED
**15.03.24**	30	CEED
**23.10.24**	38.8	CEED
**01.11.24**	23.8	IIDP
**15.11.24**	32.5	IIDP
**16.12.24**	29.5	IIDP

Supplementary Table 3. Human islet donor details. Donors of mixed sex, including 3 males (aged 34–71) and 4 females (aged 23–64). Cause of death include stroke and head trauma.

Gene	Species	Fwd (5’→3’)	Rev (5’→3’)
HK1	rat	CCAAGCGTCTCCATAAGGCA	GTTGGTCAGCCAGACGGTAA

mitochondrial tRNA^{Trp}	rat	GTGAAATCAACAACCCGCCC	ATAGTCACCCCCAGGACGAA
SPHKAP	mouse	CCAAACTCTGCTACCTGCCTT	GTGATTCCACGTTGCTGGATT
Actb	mouse	CACTGTCGAGTCGCGTCC	TCATCCATGGCGAACTGGTG

Supplementary Table 4. qPCR primer sequences.

References

1. Neelankal John A, Ram R, Jiang FX. RNA-Seq Analysis of Islets to Characterise the Dedifferentiation in Type 2 Diabetes Model Mice db/db. *Endocr Pathol* **29**, 207-221 (2018).

2. Mahajan A*, et al.* Multi-ancestry genetic study of type 2 diabetes highlights the power of diverse populations for discovery and translation. *Nat Genet* **54**, 560-572 (2022).

3. Suzuki K*, et al.* Genetic drivers of heterogeneity in type 2 diabetes pathophysiology. *Nature* **627**, 347-357 (2024).

4. Locke AE*, et al.* Genetic studies of body mass index yield new insights for obesity biology. *Nature* **518**, 197-206 (2015).

5. Pulit SL*, et al.* Meta-analysis of genome-wide association studies for body fat distribution in 694 649 individuals of European ancestry. *Hum Mol Genet* **28**, 166-174 (2019).

6. Means CK*, et al.* An entirely specific type I A-kinase anchoring protein that can sequester two molecules of protein kinase A at mitochondria. *Proc Natl Acad Sci U S A* **108**, E1227-1235 (2011).

7. Akabane S*, et al.* PKA Regulates PINK1 Stability and Parkin Recruitment to Damaged Mitochondria through Phosphorylation of MIC60. *Mol Cell* **62**, 371-384 (2016).

8. Garcia Casas P*, et al.* Simultaneous detection of membrane contact dynamics and associated Ca(2+) signals by reversible chemogenetic reporters. *Nat Commun* **15**, 9775 (2024).
