# Supplementary material for: GLP-1R associates with VAPB and SPHKAP at ERMCSs to regulate β-cell mitochondrial remodelling and function
Source: Nat Commun. 2025 Dec 10;16:11010. doi: 10.1038/s41467-025-66115-x (PMC12696101; doi:10.1038/s41467-025-66115-x)
Supplement: Supplementary file 2 — Description of Additional Supplementary Files [file 41467_2025_66115_MOESM2_ESM.pdf]

Title: Supplementary Movie 1

Description: Time-lapse confocal microscopy imaging of SNAP/FLAG-hGLP-1R and EGFP-VAPB localisation in response to exendin-4 stimulation in INS-1 832/3 SNAP/FLAG-hGLP-1R cells. SNAP/FLAG-hGLP-1R, red; EGFP-VAPB, green; images taken every 6 seconds, shown at 5 frames per second.

Title: Supplementary Movie 2

Description: Time-lapse confocal microscopy imaging of SNAP/FLAG-hGLP-1R and ERMCS localisation in response to exendin-4 stimulation in INS-1 832/3 SNAP/FLAG-hGLP-1R cells. SNAP/FLAG-hGLP-1R, red; ERMCS, SPLICS Mt-ER Long P2A, green; images taken every 6 seconds, shown at 5 frames per second; acquisition starts at 60 seconds post-exendin-4 exposure.

Title: Supplementary Movie 3

Description: Z-stack EM tomogram slices from CLEM data in Fig. 3e. Data used for image segmentation and 3D rendering shown in Fig. 3f.

Title: Supplementary Movie 4

Description: Time-lapse confocal microscopy imaging of Drp1 and mitochondria localisation in vehicle-exposed INS-1 832/3 cells. Drp1, mCherry-Drp1, Imperial College London imperial.ac.uk red; mitochondria, mito-BFP, blue; images taken every 6 seconds, shown at 5 frames per second; acquisition starts at 60 seconds post-vehicle exposure.

Title: Supplementary Movie 5

Description: Time-lapse confocal microscopy imaging of Drp1 and mitochondria localisation in exendin-4-stimulated INS-1 832/3 cells. Drp1, mCherry-Drp1, red; mitochondria, mito-BFP, blue; images taken every 6 seconds, shown at 5 frames per second; acquisition starts at 60 seconds post-vehicle exposure.
