# Supplementary material for: GLP-1R associates with VAPB and SPHKAP at ERMCSs to regulate β-cell mitochondrial remodelling and function
Source: Nat Commun. 2025 Dec 10;16:11010. doi: 10.1038/s41467-025-66115-x (PMC12696101; doi:10.1038/s41467-025-66115-x)
Supplement: Supplementary file 8 — Reporting Summary [file 41467_2025_66115_MOESM8_ESM.pdf]

nature portfolio

Corresponding author(s): Alejandra Tomas

Last updated by author(s): Oct 22, 2025

Reporting Summary

Nature Portfolio wishes to improve the reproducibility of the work that we publish. This form provides structure for consistency and transparency in reporting. For further information on Nature Portfolio policies, see our Editorial Policies and the Editorial Policy Checklist.

Statistics

For all statistical analyses, confirm that the following items are present in the figure legend, table legend, main text, or Methods section.

n/a	Confirmed	
☐	☒	The exact sample size (*n*) for each experimental group/condition, given as a discrete number and unit of measurement
☐	☒	A statement on whether measurements were taken from distinct samples or whether the same sample was measured repeatedly
☐	☒	The statistical test(s) used AND whether they are one- or two-sided<br>*Only common tests should be described solely by name; describe more complex techniques in the Methods section.*
☐	☒	A description of all covariates tested
☐	☒	A description of any assumptions or corrections, such as tests of normality and adjustment for multiple comparisons
☐	☒	A full description of the statistical parameters including central tendency (e.g. means) or other basic estimates (e.g. regression coefficient) AND variation (e.g. standard deviation) or associated estimates of uncertainty (e.g. confidence intervals)
☐	☒	For null hypothesis testing, the test statistic (e.g. *F*, *t*, *r*) with confidence intervals, effect sizes, degrees of freedom and *P* value noted<br>*Give P values as exact values whenever suitable.*
☒	☐	For Bayesian analysis, information on the choice of priors and Markov chain Monte Carlo settings
☒	☐	For hierarchical and complex designs, identification of the appropriate level for tests and full reporting of outcomes
☒	☐	Estimates of effect sizes (e.g. Cohen's *d*, Pearson's *r*), indicating how they were calculated

Our web collection on statistics for biologists contains articles on many of the points above.

Software and code

Policy information about availability of computer code

Data collection	Spinning disk confocal microscopy data was acquired using Metamorph software (Molecular Devices). Confocal data from Leica microscopes was acquired with the Leica LAS-X software. Zeiss confocal data was acquired in ZEN Blue v3.2 (Zeiss) software.

Data analysis	MS data was processed using the MaxQuant software platform (v1.6.10.43), with database searches carried out by the in-built Andromeda search engine against the Swissprot Rattus Norvegicus database (Downloaded – 21st May 2022, entries: 8,132) concatenated with the human GLP-1R protein sequence. Untargeted label-free quantitation (LFQ) intensity values, normalised to hGLP-1R levels in each immunoprecipitated sample, were analysed by LFQ Analyst. Pathway enrichment analysis with GLP-1R binding partners exhibiting changes between vehicle and exendin-4-stimulated conditions using gProfiler. Confocal and TEM image analysis was performed in Fiji. All statistical analyses and graph generation was performed using GraphPad Prism 10.2.1 (GraphPad Software Inc). Room temperature CLEM: EM tomograms were reconstructed with the IMOD software package. Correlation of confocal images and tomogram slices was done with the EC-CLEM plugin in Icy. Segmentation of the tomograms was performed manually with Microscopy Image Browser. 3D rendering of the segmentation was performed with ORS Dragonfly (www.theobjects.com/dragonfly/index.html). Cryo-CLEM: Confocal images were processed with LSM Plus (linear Wiener deconvolution). Airyscan images were processed using the Airyscan joint deconvolution algorithm; the FIB-SEM coordinate system was aligned with respect to the LSM dataset using the ZEN software (Zeiss). The FIB-SEM data was manually segmented in Fiji. Outlines were saved as masks, and 3D reconstruction performed in Icy. Q-PCR primers were designed using NCBI Primer Blast.<br>Room Temp CLEM: Tomograms were reconstructed with the IMOD software package. Correlation of confocal images and tomogram slices was done with the EC-CLEM plugin in Icy. Segmentation of the tomograms was performed manually with Microscopy Image Browser. 3D rendering of the segmentation was performed with ORS Dragonfly (www.theobjects.com/dragonfly/index.html).<br>Cryo-CLEM: the FIB-SEM coordinate system was aligned with respect to the LSM dataset using the ZEN software (Zeiss). 3D reconstruction performed in Icy.

For manuscripts utilizing custom algorithms or software that are central to the research but not yet described in published literature, software must be made available to editors and reviewers. We strongly encourage code deposition in a community repository (e.g. GitHub). See the Nature Portfolio guidelines for submitting code & software for further information.

Data

Policy information about availability of data

All manuscripts must include a data availability statement. This statement should provide the following information, where applicable:

- Accession codes, unique identifiers, or web links for publicly available datasets
- A description of any restrictions on data availability
- For clinical datasets or third party data, please ensure that the statement adheres to our policy

All data generated during the current study are included in this manuscript and/or its supplementary information files. Proteomics data is deposited in the ProteomeXchange (PRIDE) repository with accession number PXD056782 (https://www.ebi.ac.uk/pride/archive/projects/PXD056782). The Source Data file included in this paper contains data from individual experiments used to generate the final figures.

Research involving human participants, their data, or biological material

Policy information about studies with human participants or human data. See also policy information about sex, gender (identity/presentation), and sexual orientation and race, ethnicity and racism.

Reporting on sex and gender	Human islets were from cadaveric donors of mixed sex. Donor information is included in the manuscript in Supplementary Table 3.
Reporting on race, ethnicity, or other socially relevant groupings	*Please specify the socially constructed or socially relevant categorization variable(s) used in your manuscript and explain why they were used. Please note that such variables should not be used as proxies for other socially constructed/relevant variables (for example, race or ethnicity should not be used as a proxy for socioeconomic status).*<br>*Provide clear definitions of the relevant terms used, how they were provided (by the participants/respondents, the researchers, or third parties), and the method(s) used to classify people into the different categories (e.g. self-report, census or administrative data, social media data, etc.)*<br>*Please provide details about how you controlled for confounding variables in your analyses.*
Population characteristics	Statistical analysis of association between random blood glucose levels (RG) and coding DNA variants from whole-exome sequencing UK Biobank European ancestry individuals' data for SPHKAP gene variants was performed in PLINK2.0 assuming an additive genetic model. The model included covariates age, sex, time since last meal (t), accounted for as t, t2 and t3, and six principal components. Individuals were excluded if they had self-reported diabetes or diabetes medication, were pregnant, or had RG >20 mmol/L. This research has been conducted using the UK Biobank Resource under Application Number 35327.
Recruitment	N/A
Ethics oversight	Our research complies with all relevant ethical regulations. Human islets were obtained from authorised islet isolation facilities at CEED and IIDP, with all ethical approvals in place and informed relative consent obtained. These islets have been refused for transplantation, have been quality-controlled, and meet specific criteria for research purposes. Mouse islets were purified from mice bred at the Central Biological Services unit of Imperial College London. All animal procedures were approved by the British Home Office under the UK animals (Scientific Procedures) Act 1986 (Project License number PP7151519 to Dr Aida Martinez-Sanchez, Imperial College London, UK) and from the local ethical committee (Animal Welfare and Ethics Review Board) at the Central Biological Services unit of Imperial College London.

Note that full information on the approval of the study protocol must also be provided in the manuscript.

Field-specific reporting

[illegible]

Life sciences study design

All studies must disclose on these points even when the disclosure is negative.

Sample size	No sample size was calculated.
Data exclusions	No data was excluded from the analysis.
Replication	The number of biological replicates per experiment is indicated in the corresponding Figure or Supplementary Figure Legends.
Randomization	For plate reader experiments, sample allocation in the multiwell plate was randomised to minimise acquisition bias.
Blinding	Blinding was performed when possible, for example for the quantification of the number of ERMCSs per mitochondria, quantification of mitochondrial morphology from TEM data, or for cell classification by mitochondrial phenotype.

Reporting for specific materials, systems and methods

We require information from authors about some types of materials, experimental systems and methods used in many studies. Here, indicate whether each material, system or method listed is relevant to your study. If you are not sure if a list item applies to your research, read the appropriate section before selecting a response.

Materials & experimental systems

n/a	Involved in the study
☐	☒ Antibodies
☐	☒ Eukaryotic cell lines
☒	☐ Palaeontology and archaeology
☐	☒ Animals and other organisms
☐	☒ Clinical data
☒	☐ Dual use research of concern
☒	☐ Plants

Methods

n/a	Involved in the study
☒	☐ ChIP-seq
☒	☐ Flow cytometry
☒	☐ MRI-based neuroimaging

Antibodies

Antibodies used

rabbit anti-VAPB; Antibodies.com; A14703; 1:5,000 (WB)
rabbit anti-SNAP tag; New England Biolabs; P93105 1:5,000 (WB)
rabbit anti-mouse SPHKAP (kind gift from Dr Nicholas Vierra, University of Utah, 36690, 1:1,000) (WB)
mouse anti-mouse SPHKAP; gift from Dr Nicholas Vierra, University of Utah; L131/17; 1:1,000 (WB)
mouse anti-Tubulin; Sigma Aldrich; T-5168; 1:2,000 (WB)
rabbit anti-HA tag; Abcam; Ab9110; 1:1,000 (WB)
rabbit anti-GFP; Proteintech; 50430-2-AP; 1:2,000 (WB)
rabbit anti-phospho-PKA Substrate (RRXS*/T*); Cell Signaling; 9624; 1:1,000 (WB)
rabbit anti-Drp1 (D6C7); Cell Signaling; 8570S; 1:1,000 (WB)
rabbit anti-phospho-Drp1 Ser616 (D9A1); Cell Signaling; 4494S; 1:1,000 (WB)
mouse anti-human Tom20 (F-10); Santa Cruz Biotechnology; sc-17764; 1:1,000 (WB)
goat anti-mouse IgG-HRP; Abcam; ab205719; 1:5,000; (WB)
goat anti-rabbit IgG HRP; Abcam; ab205718; 1:2,000 (WB)
mouse anti-mouse GLP-1R; DSHB; Mab 7F38; 5 ug/ml (PLA)
rabbit anti-VAPB; Antibodies.com; A14703; 1:100 (PLA)
rabbit anti-mouse SPHKAP; gift from Dr Nicholas Vierra, University of Utah; 36690; 1:100 (PLA)
mouse anti-human GLP-1R; DSHB; Mab 3F52; 5 ug/ml (PLA)
rabbit anti-human SPHKAP; Atlas Antibodies; HPA042499; 1:500 (PLA)
guinea pig anti-insulin; Dako Agilent Technologies; IR002; N/A (PLA)
goat anti-guinea pig Alexa Fluor 647; Life Technologies; A21450; 10 ug/ml (PLA)

Validation

Most antibodies are commercially validated apart from:
mouse anti-mouse SPHKAP; gift from Dr Nicholas Vierra, University of Utah; L131/17 - PMID: 37758930; PMID: 37633939
rabbit anti-mouse SPHKAP; gift from Dr Nicholas Vierra, University of Utah; 36690 - PMID: 37633939

Eukaryotic cell lines

Policy information about cell lines and Sex and Gender in Research

Cell line source(s)	INS-1 832/3 cells: RRID CVCL_ZL55; Rattus norvegicus male derived insulinoma cell line.
Authentication	No authentication was performed
Mycoplasma contamination	Cell lines were tested for mycoplasma contamination monthly
Commonly misidentified lines (See ICLAC register)	N/A

Animals and other research organisms

Policy information about studies involving animals; ARRIVE guidelines recommended for reporting animal research, and Sex and Gender in Research

Laboratory animals	Mice were exclusively used for islet extraction. No in vivo procedures were performed in the study. Mice used were C57BL/6J wildtype or GLP-1R KO (generated in house, El Eid et al., bioRxiv 2024.10.19.619191).
Wild animals	N/A
Reporting on sex	Mice used for islet extraction were of mixed sex origin
Field-collected samples	N/A
Ethics oversight	Mice were bred under the UK animals (Scientific Procedures) Act 1986 Project License number PP7151519 assigned to Dr Aida Martinez-Sanchez, Imperial College London, UK; with approval from the local ethical committee (Animal Welfare and Ethics Review Board) at the Central Biological Services unit of Imperial College London.

Note that full information on the approval of the study protocol must also be provided in the manuscript.

Clinical data

Policy information about clinical studies

All manuscripts should comply with the ICMJE guidelines for publication of clinical research and a completed CONSORT checklist must be included with all submissions.

Clinical trial registration	N/A
Study protocol	N/A
Data collection	N/A
Outcomes	N/A

Plants

Seed stocks	N/A
Novel plant genotypes	N/A
Authentication	N/A
